# Exosomes contribution in COVID-19 patients’ treatment

**DOI:** 10.1186/s12967-021-02884-5

**Published:** 2021-05-31

**Authors:** Loubna Mazini, Luc Rochette, Gabriel Malka

**Affiliations:** 1 Institut Superieur des Sciences Biologiques et Paramedicales, Université Mohammed VI Polytechnique, Lot 660, 43150 Ben-Guerir, Morocco; 2 Equipe D’Accueil (EA 7460), Physiopathologie Et Epidémiologie Cérébro-Cardiovasculaires (PEC2), Faculté Des Sciences de Santé, Université de Bourgogne-Franche Comté, 7 Bd Jeanne d’Arc, 21000 Dijon, France

**Keywords:** COVID-19, Adipose derived stem cells, Mesenchymal stem cells, Exosomes, Inflammation, Patient safety

## Abstract

Adipose cell-free derivatives have been recently gaining attention as potential therapeutic agents for various human diseases. In this context, mesenchymal stromal/stem cells (MSCs), adipocyte mesenchymal stem cells (Ad-MSCs) and adipose-derived stem cells (ADSC) possessing potent immunomodulatory activities are proposed as a therapeutic option for the treatment of coronavirus disease 2019 (COVID-19). The COVID-19 represents a global concern of public health caused by severe acute respiratory syndrome coronavirus 2 (SARS-CoV-2) in which there is not actually any specific therapy. MSCs exert an immunomodulation effect due to the secretion of endogenous factors, such as vascular endothelial growth factor (VEGF), insulin growth factor (IGF), and nerve growth factor (NGF), transforming growth factor (TGF)-β and growth differentiation factor (GDF)-11. Recent reports are promising for further studies and clinical applications of ADSCs and Ad-MSCs in COVID-19 patients. Experimental and clinical studies are exploring the therapeutic potential of both MSCs and derived-exosomes in moderating the morbidity and mortality of COVID-19. In this field, more preclinical and clinical studies are warranted to find an effective treatment for the patients suffering from COVID-19 infection.

## Background

Since the SARS-CoV-2 infection has been declared as a pandemic coronavirus disease 2019 (COVID-19) by the World Health Organization (WHO) in February 2020, the disease has widely spread over more than 200 countries and represents a global concern of public health as simply human-to-human transmission via respiratory droplets. From Wuhan in China, to worldwide, more than 59,730,774 Coronavirus cases, 1,405,753 deaths and 41,322,917 recovered reported on November 24, 2020 by https://www.worldometersinfo.coronavirus.

A subset of patients with severe COVID-19 develop profound inflammation and multi-organ dysfunction consistent with a “Cytokine Storm Syndrome” (CSS). The COVID-19 induces excessive host immune responses always accompanied by a cytokine storm leading to an acute respiratory distress syndrome (ARDS) and acute lung injury followed by multiple organ failure and death. In the severe or critical COVID-19, extensive pulmonary inflammation, pulmonary edema and mild pulmonary fibrosis and consolidation were observed and largely excessed those in SARS infection [1]. There is no anti-viral effective therapy.

The virus incubation ranges from 2–14 days [2, 3]. Even some patients may be asymptomatic, fragile people are severely affected and progress to a severe ARDS with a high mortality rate [4, 5]. Lung X-ray images or computed tomography associated to hematological and biochemical blood parameters show the patient’ response to the infection but also the progression of the virus replication targeting organ homeostasis and resulting on the cytokine storm. There are evidences on a dramatic dysregulation of the host immune response against the CoV-SARS-2 involving monocytes/macrophages and T lymphocytes [6–8].

The sequences of 2019-nCoV (SARS-Cov-2) were almost identical and share 79.6% sequence identity to SARS-CoV. The both viruses share similar cell entry receptor-angiotensin (Ang) converting enzyme II (ACE2) [4]. ACE2 is a key modulator of the renin-angiotensin system (RAS), which is a signaling pathway involved in the regulation of vascular function. The major substrate of ACE2 is Ang II, which upon C-terminus cleavage, produces angiotensin 1–7 (Ang1-7) [9]. ACE2 is present on the cell surface of many tissues including the renal, cardiac, pulmonary and gastrointestinal systems, thus running an explication for the specific symptoms associated to the COVID-19. The receptor-binding domain on the surface subunit S1 of the S protein is responsible for attachment of the virus to ACE2. After binding, the S protein is cleaved at the S1/2 and S2′ regions by the transmembrane serine protease TMPRSS2: S protein priming, which in turn facilitates the fusion of the viral membrane with the membrane of the host cell and direct entry of the virus into the cytoplasm of the cell. SARS-CoV-2 uses the SARS-CoV receptor ACE2 for entry and the serine protease TMPRSS2 for S protein priming. Efforts are focused on the blockade of the virus evolution. The ACE2/COVID-19 pathway was earlier considered as a promising therapeutic target. Also, the protease TMPRSS2 being involved in the cell entry, has optionally suggested the use of the TMPRSS2 inhibitor for clinical use, thus blocking the virus entry [10]. An antiviral drug, camostat mesylate was used at that issue, where favipiravir and remdesivir were suggested based on their therapeutic action on the influenza viruses and Ebola virus, respectively [11]. Currently, more strategies are investigated on the immune therapeutic approaches including the monoclonal antibody tocilizumab targeting the IL-6 receptor [12], IL-1β blockade (anakinra) and Janus Kinase inhibition [6]. However, an increasing and rapid interest on the beneficial therapeutic effect of mesenchymal stem cells (MSCs) and adipose derived stem cells (ADSCs) in the case of the COVID-19 leading to different clinical trials investigations. ADSCs-based cell therapy products have demonstrated optimal efficacy and efficiency in different inflammation-associated diseases and ARDS for both autologous and allogeneic purposes [13–16]. Moreover, exosomes derived from MSCs and especially ADSCs have been recently gaining attention as potential therapeutic agents for various human diseases especially for immunomodulation strategies.

## COVID-19 infection and immunological manifestations

Sars-Cov2 and ACE2 interactions are the main key in occurrence of the COVID-19 symptoms. However, viral infection is more likely produced by the aberrant immune responses by IFN impairment contributing thus to the pathogenesis of the disease [17]. Chen et al. have reported that the nucleocapsid (N) protein of SARS-CoV repressed IFN-β production induced by the retinoic acid-inducible gene I (RIG-I) pathway as the key panel recognition receptor (PRR) involved in identification of RNA viruses [18]. This lead the virus evading the host innate immune response, phagocytosis, proinflammatory cytokine release and replicate efficiently, spreading thus the viral infection. The increase in neutrophil counts, D-dimer, alanin aminotransferase, total bilirubin, lactate dehydrogenase, ferritin and procalcitonin, prolonged prothrombin time, decrease of lymphocytes number and albumin were associated to severe cases [6, 8]. Also, severe lymphocytopenia with hyperactivated T cells, decreased in regulatory T cells counts with interstitial infiltering monocytes and macrophages are mostly reported [19–21]. Additionally, the release of pro-inflammatory cytokines such as IL-6, IL-1β, IL-8, and TNF-α leads to endothelial cells and epithelial lung damages [8, 22, 23]. Lymphocytopenia of CD8+ T cells and CD4+ expressing IFN-γ also occurred [24].

In severe cases, cytokine storm occurs and levels of IFN-γ, induced protein 10 (IP10), monocyte chemoattractant protein-1 (MCP-1), granulocyte-colony stimulating factor (G-CSF), tumor necrosis factor-α (TNF-α) and IL-1β were significantly enhanced [8, 23, 25]. The increase in cytokines and chemokines’ secretion also lead to the aggravation of the disease. In the ARDS-associated lung injury, systemic inflammation with pulmonary invasion and accumulation of neutrophil cells and macrophage into the alveolar spaces occurred. The absence of IFN-β and low IFN-α production and activity is related to high virus infection and inflammatory responses [26]. Autoantibodies neutralizing the ability of the corresponding type I IFNs to block SARS-CoV-2 infection were reported in patients on the onset of the COVID-19 [27]. This impairment of IFN phenotype seems to result from inborn errors of TLR3- and interferon regulatory factor 7 (IRF7)-dependent type I IFN immunity accounting for life-threatening COVID-19 pneumonia [26, 28]. Additionally, the ratio of IL-6/IFN-γ was associated to the severity of the disease [29] expecting that ADSCs would play a critical role in the immune responses by modulating their exosomes secretion containing the inflammatory cytokines mainly IL-6, IL-1β and TNF-α.

According to the dysregulation of host immune responses in the development of cytokine release syndrome (CRS) as pathologic keystone for disease evolution of Covid‐19, inhibition of IL‐6 may be a novel target for therapeutics in patients with Covid‐19. Recent findings support the requirement for the control of new clinical studies to clarify the role of immunomodulation, precisely via IL‐6 inhibition, in the cure Covid‐19 [22]. Sarilumab, Siltuximab, Tocilizumab are human monoclonal anti- bodies inhibiting the IL-6 pathway by binding and blocking the IL- 6 receptor. They may be a new therapeutic strategy for treatment of COVID-19 patients, however currently further data from large trials are required to determine whether IL-6 antagonists can provide clinical benefit in COVID-19 patients [30]. Several pharmacological approaches including glucocorticoids, inhaled nitric oxide, antioxidants and protease inhibitors have been found to be ineffective. At present, there is no specific treatment for SARS-CoV-2 infection that demonstrated an efficacy in randomized controlled trials thus, it appears urgent to test new therapies.

## Adipose derived stem cells (ADSCs) and their exosomes therapeutic use

ADSC, an abundant type of MSCs, possessing potent regenerative and immunomodulatory activities are proposed as a therapeutic option for the treatment of COVID-19. ADSCs are known to proliferate and differentiate into various cells to repair damaged or dead cells, but also act by an autocrine and paracrine pathway to activate cell regeneration and the healing process [14, 31]. They have the advantage of being abundant, easy to obtain and especially less immunogenic due to their low expression of the major histocompatibility complex I (MHC I) molecules and the lack of the MHC II and costimulatory molecules expression.

ADSCs have immune-modulating proprieties mediated by transforming growth factor-β (TGF- β), growth development factor-11 (GDF-11), hepatocyte growth factors (HGF), nerve growth factor (NGF), insulin growth factor (IGF), Interleukin-1 (IL-1), IL-6, toll like receptor (TLR)-2, TLR-4, interferon-γ (INF-γ), and a panel of miRNAs altogether secreted within their exosomes [13, 14]. ADSCs represents many therapeutic challenges in terms of origin, type, and the manner to use them, different recent investigations pave the way to their successful therapeutic use in tissue repair. ADSCs were used in many clinical investigations at both autologous and allogenic settings use and specifically for the treatment of inflammatory diseases or diseases-associated inflammation thanks to their regenerative and protective capabilities becoming thus a real issue in the case of the COVID-19 [13, 32]. It has been shown the ADSCs may be more immunosuppressive than bone marrow (BM) – or umbilical cord (UC)-MSCs. They are able to modulate immune responses through their cellular network and exosomes secretion, driving thus their surrounding microenvironment to prevent inflammation, apoptosis and senescence [14, 33].

Failure of anti-inflammatory control mechanisms within adipose tissue and peripheral blood mononuclear cells (PBMCs) have been implicated in disease progression. More importantly, ADSCs can distinguish between M1 and M2 macrophages and inducing their polarization into the anti-inflammatory profile M2 [34]. Also, ADSCs ensure the inactivation of T cells through secreting IL-10, TGF-β and impair B lymphocytes function [35]. The T-reg cell number is affected in COVID-19 patients, which explains in part severe inflammation and lung damage observed in seriously ill COVID-19 patients. Nevertheless, the increase in T-reg make them the appropriate candidate in designing treatment of COVID-19 and especially the severe cases. Therapies that enhance T-reg cell number in vivo or use T-reg-cell-derived molecules would benefit severely ill COVID-19 patients. Cytotoxic T-lymphocyte-associated protein 4 (CTLA-4) plays a critical role in T-reg-cell-mediated suppression by interacting with CD80/86 on antigen-presenting cells [36].

MSCs have attracted attention based on their multitude of functions including anti-inflammatory effects and many studies were evoked. These cells have been shown to migrate directly to the lung and partially to the liver after infusion through the tail vein in a mouse model, and this characteristic would be helpful in designing the route to administer these cell products [37]. Previous studies have confirmed their beneficial therapeutic effect in the treatment of ARDS and after E.coli endotoxin-induced acute lung injury [15, 38, 39]. The Table 1 summarizes the overall clinical trials using MSCs from different sources and/or their exosomes in treating COVID-19 patients.

**Table 1:** Mesenchymal stem cells (MSCs) collected from bone marrow (BM), umbilical cord (UC), Wharton jelly (WJ), adipose tissue (ADSCs), dental pulp (DP) or manufactured biological preparations of MSCs are administered to COVID-19 patients

Clinical trial status	Study objective	MSC origin used/mode of administration	Dose or total administered cells	Phase	Locations
Completed	Safety and efficacy Pneumonia Cytokine syndrome	UC-MSC/I.V BM-MSC/I.V ADSC/I.V	3 × 10^6^/kg or 5 × 10^7^–4 × 10^8^ cells	1/2	USA, Japan, Turkey, China, Pakistan, Indonesia
	Safety and efficacy Pneumonia	Exosomes inhalation	0.5–2 × 10^10^ nanoparticles	1/2	Russian, China
Recruiting		BM-MSC/I.V WJ-MSC/I.V UC-MSC/I.V ADSC/I.V Biological MSC/I.V DP-MSC/I.V Cryopreserved placenta derived-MSC/I.V Biological preparations of MSCs	1,5 × 10^6^–9 × 10^6^/kg or 1 × 10^8^–7.5 × 10^8^ cells	1/2	Spain, USA, China, Jordan, Mexico, Indonesia, Ukraine, Colombia, UK, Canada, Pakistan, Australia, Turkey
	Cytokine syndrome	Secretome/intramuscular	1CC/12 h × 3 days	2	Indonesia
	Pneumonia	BM-MSC + exosomes/I.V	2 × 10^8^ + Exosome dose (NA)	2/3	Iran
Not yet recruiting	Pneumonia Efficacy	UC-, BM-, ADSCs-, DP-MSCs, biological preparations/I.V	5 × 10^5^–2 × 10^7^/Kg or 8 × 10^7^–6 × 10^8^ cells	1/2	Brazil, China, USA, Germany
	Viral inflammation Pneumonia	MSCs derived exosomes (ARDOXO)/ I.V UCB educator and MSCs secretome/ IV	2–8 × 10^9^/ml 1 × 10^6^/Kg or conditioned media	1/2	USA
Active not recruiting	Pneumonia Safety and efficacy	WJ-, UCB-MSCs, ADSCs/ I.V	4 × 10^6^/Kg or 8 × 10^7^–8 × 10^8^ cells	1/2	Mexico, USA, China, Spain

Route of administration, doses and selection criteria of patients are different according to the objectives and the status of the clinical trial. Purified exosomes and MSCs derived secretome are also administered in patients alone or associated to MSCs

*I.V* intravenous infusion, *NA* not applicable

https://www.clinicaltrials.gov

**Fig. 1:**
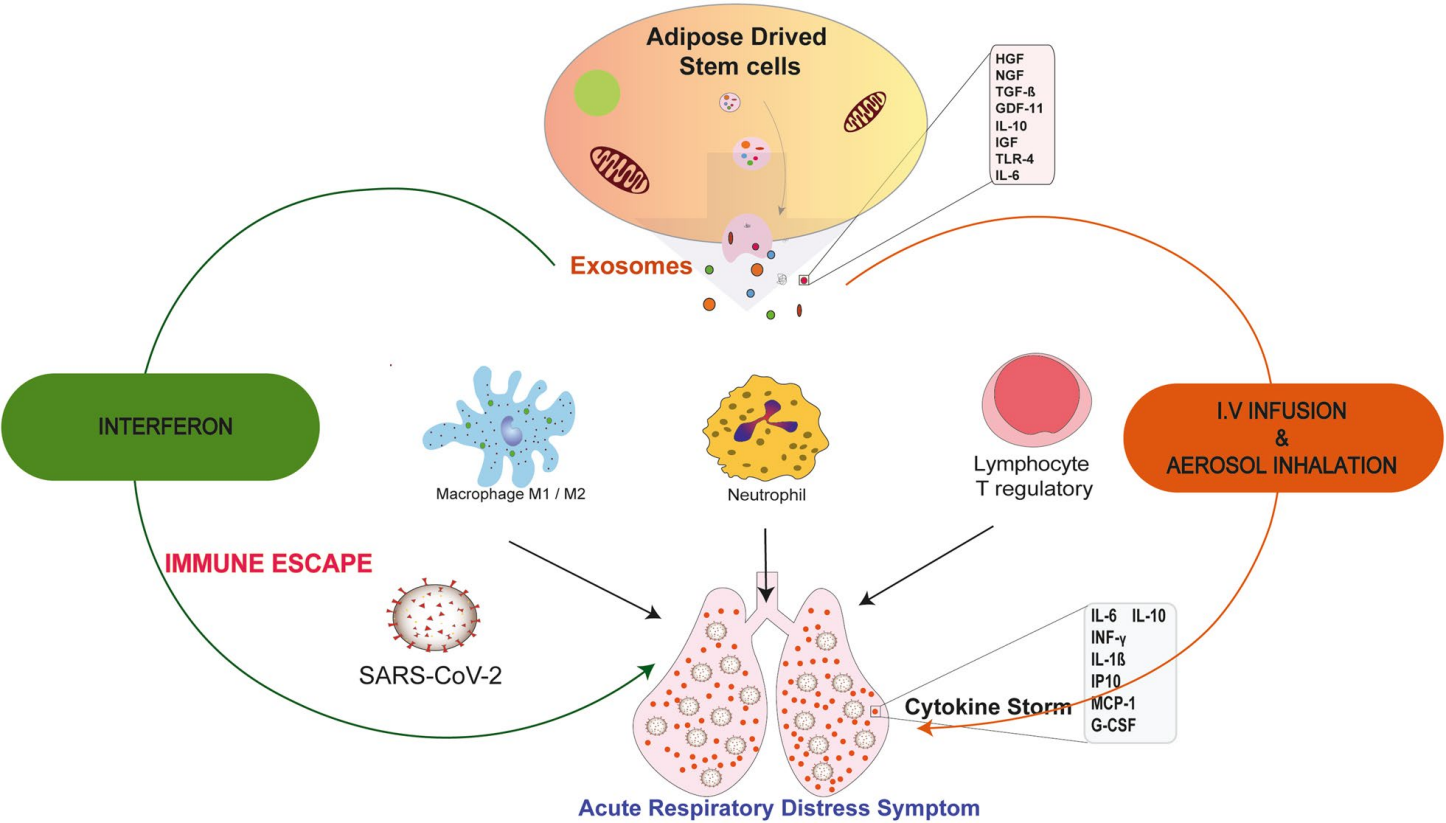
Exosomes released from Adipose derived stem cells (ADSCs) secrete a panel of growth factors involved in reducing the cytokine storm and rescuing patients from the acute respiratory distress symptoms through the increase in neutrophils and T reg numbers and the polarization of macrophages from M1 to M2 profile. HGF: hepatocyte growth factor, NGF: nerve growth factor, IGF: insulin growth factor, GDF-11: growth differentiation factor-11, IL-10: interleukin-10, TLR-4: Toll like receptor, TGF-β: transforming growth factor-β, TNF-α: tumor growth factor-α, MCP-1: monocyte chemoattractant protein-1, IP-10: induced protein 10, G-CSF: granulocyte colony stimulating factor

Interestingly, MSCs are not infected by the coronavirus [40]. Systemic administration of 2 × 10^6^ cells MSCs/kg resulted in the reduction of multiple pulmonary and systemic markers of inflammation, epithelial cells apoptosis, alveolar-capillary fluid leakage, and proinflammatory cytokines while the suppression of T-cell responses and induction of regulatory phenotypes in T cells, monocytes, and neutrophils have proven the anti-inflammatory induction of these cells against ARDS when the standards clinical measures have failed (Fig. 1) [39]. Additionally, intravenous infusion has proven more efficiency in different clinical trials. To search for potential factors that may influence the design of new trials, data on routes of administration were evaluated [41].

In addition, ADSCs derived exosomes were involved in many biological functions, such as cell proliferation, immune regulation, and intercellular communications in normal and pathophysiological processes [42, 43]. They have proven their efficiency in promoting cell proliferation, migration, angiogenesis, limiting cell apoptosis, reducing inflammation and oxidative stress and participating to the immune regulation [44–47]. In contrary to MSCs, exosomes have the advantage to migrate to the target organs instead of being trapped by the lung and hence may be approved as a therapeutic tool through aerosol inhalation applications [48, 49]. Derived from ADSCs, exosomes have already proven efficiency in immune responses by increasing T cell regulator leading to the increase of anti-inflammatory IL-4 and IL-10 levels associated to the decrease of the proinflammatory cytokine levels IL-17 and IFN. This immunomodulatory effect was benefit in modulating T-cell inflammatory response in autoimmune diabetes type 1 and atopic dermatitis [50, 51]. These exosomes have the ability to activate M2 macrophage polarization reducing thus inflammation [52]. Additionally, exosomes were reported to increase the neutrophil number and viability while reducing eosinophils and infiltering mast cells suggesting their potential benefit in infections and immunodeficiency diseases [53]. At the other side, exosomes contain a panel of micro-RNAs (miR) including miR-21, miR-23a, and miR-124 recognized as having an immunosuppressive and anti-inflammatory role [54]. These findings pave the way to the several approaches performed to ensure bioavailability of safe and efficient exosomes derived from ADSCs and BM-MSCs as drug delivery systems for immunomodulatory investigations [55, 56].

Currently, many clinical trials were investigated using ADSCs. Seven studies are registered for MSCs derived from BM, 17 using UC-MSCs including 5 not yet recruiting assays, 7 recruiting and 2 actives not recruiting trials (Table 1). Ten clinical trials using autologous and allogenic ADSCs were registered with 4 not yet recruiting, 3 recruiting and 3 are active not recruiting. Mostly performed in the USA, China and Spain, these trials aimed safety and efficacy of treated patients and to provide protection against COVID-19 https://clinicaltrials.gov/ct2/results?cond=COVID,19&term=Mesenchymal%20Stem%20Cells&cntry=&state=&city=&dist= . Manufactured biological MSCs from BM, UC and adipose tissue are differently prepared such as NestaCell, Astrostem, Prime Pro, Descartes 30, among others. The registered trials are mostly phase I, II or phase I/II and few are phase II/III performed with MSCS intravenous administration with doses ranging from 1 × 10^6^ cells/kg to 3 × 10^6^ cells/Kg or 5 × 10^7^ to 8 × 10^8^ total cells as potential therapy for ARDS associated to different etiologies including the COVID-19. However, 2 clinical trials advocate the use of 3–4 × 10^7^ MSCs to treat the COVID-19-associated ARDS [57]. After intravenous administration of clinical-grade ACE2-mesenchymal stem cells, an interesting improvement in pulmonary functional activity was shown 2 days after in patients affected by COVID-19 accompanied with a significant decrease ratio of serum pro-inflammatory cytokine TNF-α.

Secondary to the studies using injection of MSCs, potential use of ADSCs and adipose-derived mesenchymal stem cells (AD-MSCs) in COVID-19 draws more attention due to their immunologic and regenerative effects. Autologous and allogenic ADSCs have already proven their safety and efficacy in several human clinical trials of immune mediated inflammatory diseases and also demonstrated in cardiovascular, pulmonary, metabolic, neurologic and immune diseases [14, 58, 59]. Actually, even clinical trials using ADSCs or derivatives are planned, clinical results are still not reported. In a randomized, placebo-controlled pilot study, allogeneic ADSCs systemic administration was performed in patients with ARDS to examine the possible adverse events, confirming thus their safety [60].

Recent reports are promising and encouraging for further studies and clinical applications of ADSCs- and MSCs-derived exosomes for the treatment of COVID-19 patients. Some clinical trials were registered and pave the way to their parent cells supported by the safety and efficiency of their preparation [58, 61, 62]. Additionally, growing promising results using these extracellular vesicles to treat a wide range of diseases is largely described [42, 63, 64]. Indeed, protocols investigations, based on the promising result of a pilot study (NCT04276987) were developed in different trials treating severe COVID-19 pneumonia (NCT04602442, NCT04491240, ChiCTR2000030261) (https://covid-19.cochrane.org/?q=k(exosomes)&pn=1) aiming to improve their safety preparations and therapeutic outcomes after exosomes inhalation. BM-MSCs derived exosomes ExoFlo® used in a prospective nonrandomized open-label cohort study also appeared safe in improving pneumonia parameters, downregulating the cytokine storm and reconstituting COVID-19 patients’ immunity [65].

In the clinical study ChiCTR2000030484, UC-MSCs and their exosomes were used intravenously to treat lung disease during the COVID-19 pneumonia at 5 × 10^7^ cells (4times) and 180 mg/time (14 times), respectively. Purified exosomes XoGlo® were administered intravenously into severe COVID-19 patients presenting requiring invasive mechanical ventilation for respiratory failure due to pneumonia or requiring treatment with vasopressors (ISRCTN33578935). The Zofin (Organicell Flow) is developed from human amniotic fluid with a mean concentration of 5.24 × 10^11^ particles/mL are tested in patients presenting COVID-19 infection with SARS. This phase I/II Randomized, Double Blinded, Placebo Trial aimed to evaluate the Safety and Potential Efficacy of Intravenous Infusion of Zofin (NCT04384445). Tsuchiya et al. have planned a study in china to investigate the effects of aerosol inhalation of 2.0 × 10^8^ nanovesicles (5 times) in treating severe COVID-19 patients [66]. T cells have also triggered attention in treating the early stage of pneumonia in COVID-19 patients, a clinical study (NCT04389385) uses exosomes derived from allogenic COVID-19 T cells and administered through aerosol inhalation. At the other side, a pilot clinical study uses allogenic ADSCs-derived exosomes to treat through inhalation pneumonia in severe patients with novel coronavirus (NCT04276987).

## Conclusion

To explore the therapeutic potential of both MSCs and ADSCs and exosomes in moderating the morbidity and mortality of COVID-19 and treating the associated ARDS, inflammatory biomarkers represented by various cytokines and D-dimers are used to identify the outcome and COVID-19 patients’ recovery. If the limitations related to the clinical use of ADSCs have been largely discussed, using exosomes raises some issues which could not be neglected. The International Society for Extracellular Vesicles (ISEV) in 2018 have recently promoted the need of standardizing culture, purification and storage protocols of exosomes [67]. Moreover, exosomes cargo remain closely associated to the functional status of their parent cells and we can expect than activated, immature and committed cells display variability in their secretory profile which can be acknowledged by the composition of their secreted exosomes [23, 64, 68]. Additionally, young MSCs-derived exosomes exceed their aged counterparts in alleviating lipopolysaccharride-induced acute lung injury [69], probably through the growth differentiation factor 11 (GDF11) largely recognized for its regenerative potential and tissue repair in different organs [33, 70–73]. These factors might lead to variability in therapeutic potency of exosomes, suggesting that additional quantitative and qualitative markers should be used to forecast the therapeutic efficacy of used exosomes in COVID-19 especially. The ISEV and the international society for cell and gene therapy have stated on the need of specific guidelines and standardizations of the operating procedures to address the production of efficient exosomes meeting the good manufacturing practices criteria [67, 74, 75]. Accordingly, we have also recently reported that refining cell-based therapies is becoming challenging in the context of the COVID-19 to fully attain efficiency and patient safety [76]. Finally, managing all these constraints in a record time remains however the big world challenge to stop this pandemic.

## Data Availability

Not applicable.
